# Assessing *CD 10* Expression Level and Its Prognostic Impact in Egyptian Patients with Urothelial Carcinoma

**DOI:** 10.31557/APJCP.2020.21.6.1573

**Published:** 2020-06

**Authors:** Marwa T Hussien, Eatemad Helmy, Tarek M Elsaba, Azza Elkady, Hani Alrefai, Helal F Hetta

**Affiliations:** 1 *Department of Pathology, South Egypt Cancer Institute, Assiut University, Assiut, Egypt. *; 2 *Department of Pathology, Faculty of Medicine, Assiut University, Assiut, Egypt. *; 3 *Sohag University Medical Administration, Sohag, Egypt. *; 4 *Department of Medical Biochemistry and Molecular Biology, Faculty of Medicine, Mansoura University Mansoura, Egypt. *; 5 *Department of Medical Microbiology and Immunology, Faculty of Medicine, Assiut University, Assiut, Egypt.*; 6 *Department of Internal Medicine, University of Cincinnati College of Medicine, Cincinnati, OH , USA. *

**Keywords:** CD10, mRNA, urothelial, stage, recurrence, metastasis

## Abstract

**Background and aim:**

CD10 is expressed in urothelial carcinoma cells and cancer associated fibroblasts (CAF). In the current study, CD10 immunohistochemical staining (IHC) and CD10 mRNA expression in urothelial carcinoma of bladder (UCB) were assessed, and its relationship with tumor progression and prognosis was investigated.

**Patients and Methods:**

In this study, 106 formalin fixed paraffin-embedded (FFPE) tissue of UCB, obtained through radical cystectomy specimen, and 10 matched normal tissue samples were included.CD10 expression was evaluated by immunohistochemistry and real time PCR techniques.

**Results:**

CD10 expression in tumor cells and associated stromal fibroblasts was significantly associated with high tumor grade and advanced stage. Significant correlation was found between CD10 tumor expression and lymphovascular invasion (LVI) (P<0.001) as well as perineural invasion (PNI). CD10 expression in stromal fibroblasts was significantly associated with squamous differentiation of tumor cells, lymph node metastasis (LNM), and tumor necrosis. Positive CD10 expression in both tumor cells and associated stromal fibroblasts was associated with shorter OS . CD10 mRNA was overexpressed in tumors in comparison with the matched normal tissues. CD10 mRNA was significantly higher in invasive tumor, advanced stage tumor, and high grade tumor. There was significant correlation between CD10 mRNA tumor expression and LVI, PNI, and tumor recurrence.

**Conclusion:**

Increased expression of CD10 in the tumor and CAF was strongly correlated with tumor progression, invasion, metastasis, shorter OS, and RFS in urothelial carcinoma patients. CD10 mRNA showed significantly higher expression in tumor tissue than in matched normal tissue. CD10 mRNA was associated with depth of invasion, TNM stage, tumor grade, vascular tumor invasion, and tumor recurrence.

## Introduction

Urinary bladder cancer ranks as the ninth most frequently-diagnosed cancer worldwide. It’s incidence rate is consistently higher in men than women, although sex differences varied greatly between countries (Antoni et al., 2017).

The rate of bladder cancer is higher in Egypt than all other countries over the world. Bladder cancer is considered to be the third most common malignancy worldwide (Elshikh et al., 2015), accounting for 8.7% of total cancer case (Fedewa et al., 2009). Nowadays, urothelial carcinoma has become the predominant histopathologic type of urinary bladder cancer in Egypt as a result of significant change in etiology of bladder cancer (Felix et al., 2008).

The future of bladder cancer management will rely on the use of validated multimarker panels for risk stratification, optimal surgical management, and therapeutic strategies to identify and target specific alterations in individual tumors (Mitra, 2016).

Enormous advances have been made in understanding the molecular basis of bladder tumorigenesis and progression. Bladder cancer is characterized by specific molecular defects, and this information is being translated to develop methods to assess cancer at the cellular and molecular levels (Noel et al., 2009).

The current study was performed on 106 cases of UCB referred to South Egypt Cancer Institute (SECI). We first investigated the expression of CD10 protein in urothelial carcinoma cells and cancer associated fibroblasts (CAF) by IHC study and evaluated the correlation between CD10 expression and different clinicopathological data and survival analysis. In addition, we quantified CD10 mRNA in urothelial carcinoma by RT-PCR in comparison with matched normal urinary bladder tissues and its correlation with various clinicopathologic data. 

## Materials and Methods

*Samples*

This study was done on formalin–fixed paraffin–embedded tissue blocks. These blocks were obtained from the South Egypt Cancer Institute. The protocol of the study was first approved by International Review Board (IRB). The martial collected in the period from January 2011 to December 2013, and followed up until December 2016 .

These paraffin blocks were obtained from radical cystectomy specimens of 106 cases with urothelial carcinoma of the bladder. Comparison with the following parameters as patient age and sex, tumor size, tumor grade and stage, papillary architecture, squamous differentiation, and bilharzial infestation. These parameters were based on histopathological reports. The tumors were graded and staged according to WHO / ISUP grading system (Cheng et al., 2012) and TNM WHO pathologic staging system of urinary bladder cancer (Fred et al., 2016), respectively.

*Procedures*

Immunohistochemistry: The formalin fixed paraffin-embedded (FFPE) tissue sections were cut with 3-5 μm thickness, and then mounted on glass slides. Obtained sections were dewaxed in Xylene and then rehydrated by graded alcohols. Dako PT Link (code PT 100/ PT101) was used for pre-treatment with heat-induced epitope retrieval (HIER). The slides were washed with PBS twice. Then, Dako peroxidase blocking reagent was applied and incubated 5 minutes at room temperature. Next, step applied Primary FLEX monoclonal mouse anti-human CD10 antibody (clone 56C6, Code IR648, Dako Denmark A/S, 117568-002) Ready–to-use , and incubated for 1 hour at room temperature. Then, a universal staining kit “Envision Detection System Anti-Polyvalent, HRP/DAB (Ready-To-Use)” (EnVision Flex, high PH, Link, code K800021-2, Dako Denmark A/S) was introduced to the slides, according to the manufacturer’s instructions, for 20 minutes at room temperature. Counter stained the section using EnVision FLEX hematoxylin (Link) (code K8008), followed by dehydration in alcohols and cleared in Xylene .

The positive control for of the CD10 specificity evaluation was obtained from healthy renal tissue. The positivity represents brown membranous and cytoplasmic staining in the cells of both glomeruli and tubules. The negative control of urothelial carcinoma tissue section was done by omitting primary antibody while using the same immunohistochemistry protocol. 

*CD10 positivity assessment*

CD10 positivity required brown staining of the cell membrane and/or cytoplasm. A 5% cut-off point in urothelial carcinoma cells and cancer associated stromal fibroblasts, separately (Abdou, 2007). 

*Real time PCR*

Specimen selection: Sixty FFPE tissue sections were processed by RT-PCR. Fifty of them were chosen randomly from the whole urothelial carcinoma specimens examined previously with immunohistochemistry. The other ten specimens were healthy urinary bladder tissues of non-tumorous cases, obtained from the registry of the Pathology Department in SECI.

RNA extraction and relative mRNA quantification

Total RNA was extracted from the FFPE tissues according to manufacturer’s protocol (company, 2014) . Then, one step RT-PCR using QuantiFast Probe RT-PCR +ROX Vial dual-labeled probes (catalog NO. 151037628, Qiagen) (campany, 2011). CD10 mRNA expression was quantified using TaqMan gene expression assay a 7500 Real-Time PCR System (Applied Biosystems) in accordance with the manufacturer’s instructions. CD10 mRNA level in the tumor was compared with that in matched normal mucosa, as an internal control gene, after standardization against GAPDH. The CD10 mRNA level was calculated using the following formula: 

– ∆∆CT = ∆CT of tumor – ∆CT of matched normal tissue

∆CT of tumor= CD10 tumor Ct - GAPDH Ct

∆CT of matched normal tissue=CD10 normal tissue Ct - GAPDH Ct

 After that, we calculated – ∆∆CT to the power of two to obtain 2−∆∆CT (fold change) or relative quantitation (Shin Fujita 2007). This value was the ratio of CD10 mRNA in the tumor relative to that in matched normal tissue (T/N). 

*Statistical Analysis*

Data are presented as numbers and percentage for qualitative data; and as median value (in abnormal distribution) and mean and standard deviation (in normal distribution) for quantitative data. A2 *χ2* chi-square test was used to assess the association between CD10 protein expression in tumor cells and CAF and other clinicpathologic features. Fissure value was used when cells counted less than 5. MannWhitney U test was used to assess the association between CD10 protein expression in tumor cells and CAF and tumor size after application of normality test. Kaplan - Meier survival test was used to analyze overall survival (OS), recurrence free survival (RFS), and distant metastasis free survival (MFS). PCR results on mRNA, which expressed as fold change, were compared with clinicopatholgical features of patients by using MannWhitney U test and Kruskal-Wallis test. Relationship between CD10mRNA of tumor specimen and CD10 IHC of both tumor cells and stromal fibroblast was statistically tested via MannWhitney U test. Kruskal-Wallis test was used to study the relation between CD10 IHC group expression and CD10mRNA of tumor. Correlation between CD10mRNA of tumor and CD10 IHC of both tumor cells, and stromal fibroblast was tested by Spearman’s rho. Results were statistically analyzed using statistical package for Social Sciences (SPSS, version 20).

## Results

*Demographic data*

Clinicopathologic data of 106 cases of urothelial carcinoma of the urinary bladder were obtained from the patient`s clinical sheets at the SECI registry. Analysis of these data revealed that 86 (81.1%) were males and 20 (18.9%) were females. Their size of tumor mass was ranged from one to twelve cm. 

**Figure 1:**
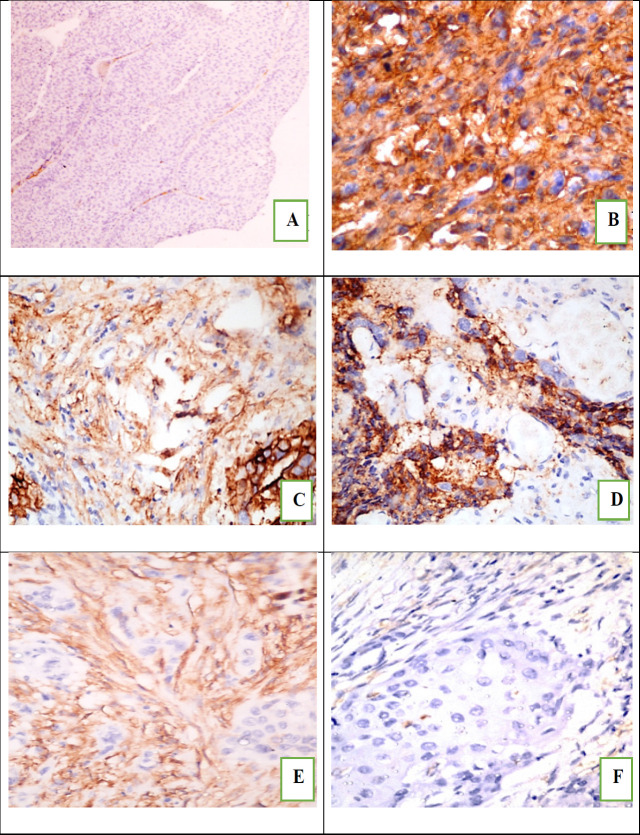
*CD10* Expression in Tumor Cells and Positive Control: (A), Non-invasive low grade uothelial carcinoma showed negative CD10 staining (low power examination); (B), Invasive high grade urothelial carcinoma showed diffuse membranous staining (high power examination); (C), Simultaneous expression of CD10 in both tumor and CAF (high power examination). (B) The tumor cells only stained with CD10 while CAF were negative (high power examination). (C) The CAF were positive for CD10 and the tumor cells were negative (high power examination); (D) Negative staining of both tumor and CAF (high power examination).

**Figure 2:**
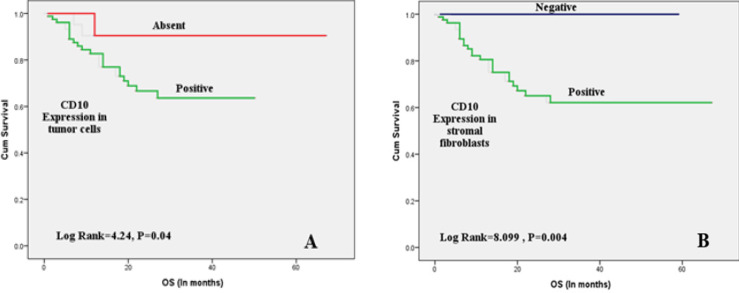
Kaplan Meier Survival Plots for OS with CD10 Protein Expression in Tumor Cells and Stromal Fibroblasts: (A)Positive CD10 expression in tumor cells was significantly associated with decreased OS. (B) Positive CD10 expression in stromal fibroblasts was significantly associated with decreased OS

This study included 15 (14.2%) cases of low tumor grade and 91 (84.8%) cases of high grade urothelial carcinomas. In all, 7 (6.6%) cases were stage pTa, 13 (12.3%) stage pT1 , 6 (5.7%) stage pT2a, 14 (13.2%) stage pT2b, 48 (45%) stage pT3, and 19 (17.9%) were stage pT4 (Figure 1-2). Squamous differentiation of tumor cells was noticed in 50 (47.1%) cases. Evidence of bilharzial infestation was detected in 43 cases (40.6%). Regarding lymph nodes affection, 30 (28.3%) cases had metastatic tumor deposits in LNs. Seventy-seven (72.6%) cases showed lymphovasular tumor emboli. perineural invasion (PNI) was detected in 71 (67%) cases. Tumor necrosis was present in 68 (64.2%) cases.

The mean OS was 21.3±16.5 months (ranging from 0 to 67 months), mean recurrence free interval (RFI) was 18.9±17.4 months (ranging from 0 to 67 months), and mean distant metastasis free interval (MFI) was 20.97±16.72 months (ranging from 1 67 months). Total number of deaths at the end of the study was 23 cases (21.7%), while 34 cases (32.4%) had local recurrence, and 10 cases (9.5%) experienced distant metastasis.

All clinicopathologic features are summarized in Table 1.

*Evaluation of CD10 expression by IHC and RT-PCR*

*Immunohistochemical finding*

Eighty-two cases (77.4%) were positive for CD10 expression in tumor cells, while 24 cases (22.6%) were negative. Positivity in CAF was present in 86 cases (81.1%), while it was absent in 20 cases (18.9%).

Association between *CD10* expression in either tumor cells or cancer associated stromal fibroblasts and clinicopathologic characteristics 

As shown in Table 2, *CD10* expression in tumor cells was significantly associated with high tumor grade (P<0.001), invasiveness of tumor, and advanced stage of the disease (P<0.001). Significant correlation was also present between *CD10* tumor expression and lymphovascular invasion (LVI) (P<0.001) and PNI (P=0.001). The association between *CD10* expression in tumor cells and patient’s sex, lymph node metastasis, tumor size, tumor necrosis, or squamous differentiation was not statistically significant. Positive correlation was present between *CD10* expression in stromal fibroblasts and high tumor grade (P<0.001), invasiveness of tumor, advanced stage (P<0.001), LVI (P<0.001), and PNI (P<0.001). Unlike* CD10* expression in tumor cells,* CD10* expression in stromal fibroblasts was significantly associated with squamous differentiation of tumor cells (P=0.023), lymph node metastasis (P=0.043), and tumor necrosis (P<0.001). The papillary architecture was negatively correlated with *CD10* expression (P<0.001). No significance correlation was found between *CD10* expression in stromal fibroblasts and patient’s sex or tumor size. 

*Survival analysis*

**Figure 3:**
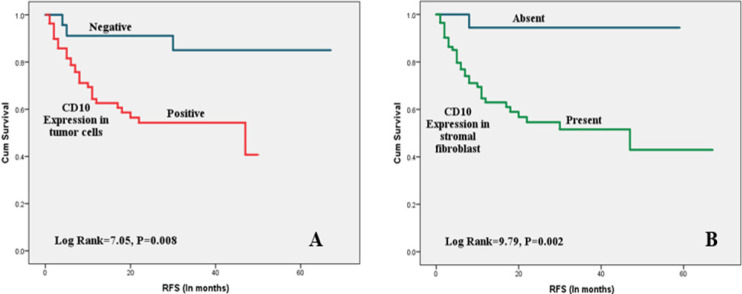
Kaplan Meier Survival Plots for RFS with CD10 Expression in Tumor Cells and Stromal Fibroblasts: (A) Positive CD10 expression in tumor cells was significantly associated with decreased RFS. (B) Positive CD10 expression in stromal fibroblasts was significantly associated with decreased RFS

**Figure 4:**
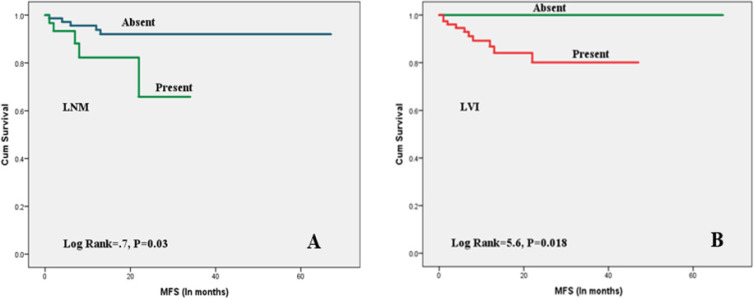
Kaplan Meier Survival plots for MFS with Different Clinicopathologic Variables (LNM and LVI): (A), LNM was significantly associated with decreased MFS; (B), LVI was significantly associated with decreased MFS

As shown in Table 3, high tumor grade, advanced stage, LVI, LNM , PNI, and positive CD10 expression in both tumor and CAF (Figure 2) were significantly associated with poor OS. High tumor grade, advanced stage, LVI, LNM, PNI, tumor necrosis, squamous differentiation, and positive CD10 staining in both tumor cells and CAF (Figure 3) were associated with shorter RFS. Only LVI and LNM were associated with shorter MFS (Figure 4).

*Relationship between CD10 mRNA expression in tumor and patients’ clinicopatholgical features*

PCR results of mRNA, which expressed as fold change, were compared with clinicopatholgical feature of patients by MannWhitney U test and Kruskal-Wallis test (Table 4).

*CD10 mRNA* expression in tumor was significantly associated with tumor grade (p<0.001) with high median fold change of high grade (7.857) in contrast to lower median fold change of low grade (1.874). There was a significant relationship between CD10 mRNA expression in tumor and tumor stage (p<0.001). Significant correlation was present between CD10 tumor expression and LVI, PNI, and tumor recurrence. However, patients’ sex, lymph node metastasis, tumor necrosis, and squamous differentiation had no statistically significant relationship with fold changes.

Despite that the median fold change was higher in metastatic group (10.996) than the non-metastatic one (6.265), there was no significant association between CD10 mRNA expression and distant metastasis. The medians fold changes in survived and dead groups were 6.184 and 9.172, respectively, revealing no significant association between CD10 mRNA expression and survival status.

*Correlation between CD10 IHC expression and CD10 mRNA expression of tumor based on PCR*

Relationship between CD10 mRNA in tumor specimen and CD10 IHC in both tumor cells and stromal fibroblast was statistically examined via MannWhitney U test (Table 5). *CD10 mRNA* expression in tumor was significantly associated with *CD10 IHC *expression in tumor cells (P <0.001). The median fold change was extremely high in positive cases (7.452) relative to negative cases (1.959) according to IHC There was also significant association between *CD10 mRNA* expression in tumor and CD10 stromal fibroblasts based on IHC (P=0.002). According to IHC, the median fold change was high in positive cases (7.452) in contrast to negative cases (2.249).

**Figure 5:**
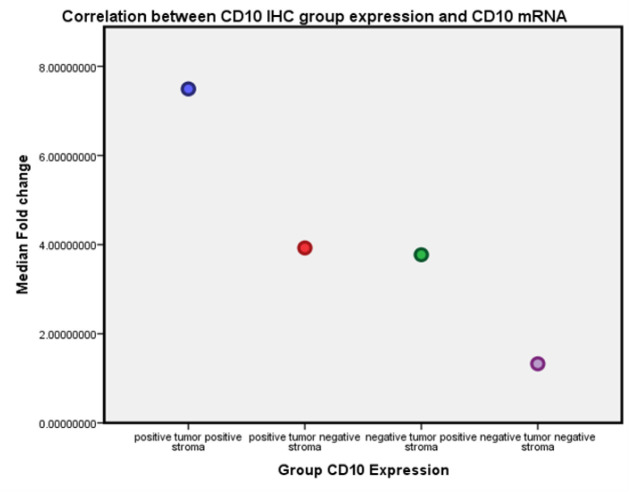
Correlation between CD10 IHC Expression Groups and mRNA in Tumor Specimen: The median fold change was higher in the first group, while lower median fold change was present in the fourth group. The second and third groups showed minimal difference in median fold changes

Kruskal-Wallis test was used to study the relation between *CD10 IHC* expression and CD10 mRNA in tumor (Table 5). A significant association between *CD10 IHC* expression and CD10 mRNA in tumor was revealed (P=0.007). The median fold change was higher in the first group, which included cases with both positive tumor and stromal cells CD10. The least median fold change was present in the fourth group, which included cases with CD10 and both negative tumor and stromal cells. The second and third groups showed minimal difference regarding median fold changes (Figure 5).

**Figure 6:**
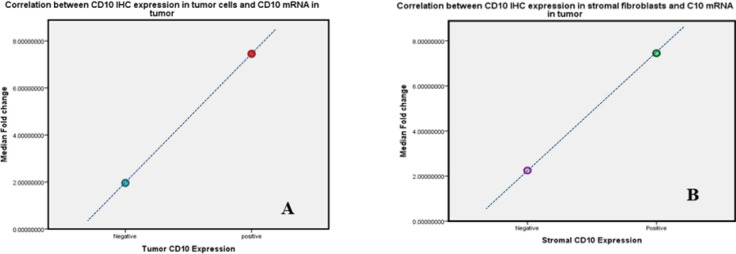
Correlation between CD10 Marker in both IHC and PCR Techniques among Cases: (A) Strong Positive Correlation between CD10 IHC Expression in tumor cells and mRNA in tumor specimen (B) Strong positive correlation between CD10 IHC expression in stromal fibroblasts and mRNA in tumor specimen

Correlation between CD10 mRNA in tumor and CD10 IHC in both tumor cells and stromal fibroblast was examined by Spearman’s rho (r) (Table 6). A significant positive correlation was found between *CD10 mRNA *and *CD10 IHC* expression in tumor cells (r=0.487, p<0.001) (Figure 6-A). In addition, a significant positive correlation was found between *CD10 mRNA* and *CD10 IHC* expression in stromal fibroblasts (r=0.440, p=0.002) (Figure 6-b).

**Table 1:** Clinicopathological Features of Patients

Parameters	NO. (%)
Sex	
Male	86 (81.1%)
Female	20 (18.9%)
Grade	
Low	15 (14.2%)
High	91 (84.8%)
Stage	
pTa	7 (6.6%)
pT1	13 (12.3%)
pT2a	6 (5.7%)
pT2b	14 (13.2%)
pT3	47 (44.3%)
pT4	19 (17.9%)
Bilharziasis	
Present	43 (40.6%)
Absent	63 (59.4%)
Squamous differentiation	
Present	50 (47.1%)
Absent	56 (52.9%)
Lymphovascular invasion (LVI)	
Present	77 (72.6%)
Absent	29 (27.4%)
Lymph node metastasis (LNM)	
Present	30 (28.3%)
Absent	76 (71.7%)
Perineural invasion (PNI)	
Present	71 (67%)
Absent	35 (33%)
Necrosis	
Present	68 (64.2%)
Absent	38 (35.8%)
Tumor Size in cm	
Range	1 to 12 cm
Median	4
Recurrence free interval in months	
Range	0 to 67
Mean ± SD	18.9 ± 17.4
Recurrence	
Present	34 (32.4%)
Absent	71 (67.6%)
Metastasis free interval in months	
Range	1 to 67
Mean ± SD	20.97±16.72
Metastasis	
Present	10 (9.5%)
Absent	95 (90.5%)
Survival time in months	
Range	0 to 67
Mean ± SD	21.3 ± 16.5
Status	
Alive	82 (77.4%)
Dead	23 (21.7%)

Data was expressed as (Mean ± SD), number and percentage; SD, Standard Deviation.

**Table 2:** Correlation between Clinicopathologic Features and CD10 Expression (Using 5% Cut off Point in Both Tumor and CAF)

Parameters	*CD10* expression in tumor cells		Test of sig.	*CD10* expression in CAF		Test of sig.
	Positive	Negative		Positive	Negative	
	No. (%)	No. (%)	P	No. (%)	No. (%)	P
Sex						
Male	65 (77.4%)	19 (22.6%)		65 (75.6%)	16 (80%)	
Female	17 (77.3%)	5 (22.7%)	P = 1.00 #	21 (24.4%)	4 (20%)	P = 0.78 #
Grade						
Low	4 (28.6%)	10 (71.4%)		3 (3.5%)	13 (65%)	
High	78 (84.8%)	14 (15.2%)	P <0.001#	83 (96.5%)	7 (35%)	P <0.001#
Stage						
pTa	0 (0%)	8 (100%)	ᵪ2 = 35.9	0 (0%)	7 (35%)	χ2 = 56.1
pT1	7 (58.3%)	5 (41.7%)	P < 0.001	4 (4.7%)	6 (30%)	P < 0.001
pT2a	4 (80%)	1 (20%)		6 (7%)	0 (0%)	
pT2b	12 (80%)	3 (20%)		13 (15.1%)	2 (10%)	
pT3	44 (91.7%)	4 (8.3%)		49 (56.9%)	1 (5%)	
pT4	15 (83.3%)	3 (16.7%)		14 (16.3%)	4 (20%)	
Bilharziasis						
Present	34 (79.1%)	9 (20.9%)	ᵪ2 = 0.67	35 (40.7%)	8 (40%)	χ2 = 0.007
Absent	48 (76.2%)	15 (23.8%)	P = 0.4	51 (59.3%)	12 (60%)	P =0.95
Squamous differentiation						
Present	43 (84.3%)	8 (15.7%)	ᵪ2 = 2.7	44 (51.2%)	4 (20%)	χ2 =6.32
Absent	39 (70.9%)	16 (29.1%)	P = 0.09	42 (48.8%)	15 (8%)	P =0.023
Lymphovascular invasion (LVI)						
Present	67 (81.7%)	10 (41.7%)	ᵪ2= 14.9	72 (83.7%)	5 (25%)	χ2 = 38.2
Absent	15 (18.3%)	14 (58.3%)	P <0.001	14 (16.3%)	15 (75%)	P< 0.001
Lymph node metastasis (LNM)						
Present	24 (29.3%)	6 (25%)	ᵪ2= 0.17	28 (32.6%)	2 (10%)	χ2 = 5.04
Absent	58 (70.7%)	18 (75%)	P= 0.68	58 (67.4%)	18 (90%)	P = 0.043
Perineural invasion (PNI)						
Present	60 (73.2%)	11 (45.8%)	ᵪ2= 6.3	65 (75.6%)	6 (30%)	χ2= 15.24
Absent	22 (26.8%)	13 (54.2%)	P= 0.01	21 (24.4%)	14 (70%)	P< 0.001
Necrosis						
Present	57 (69.5%)	11 (45.8%)	ᵪ2= 0.03	62 (72.1%)	6 (30%)	χ2= 12.56
Absent	25 (30.5%)	13 (54.2%)	P = 4.5	24 (27.9%)	14 (70%)	P< 0.001
Tumor Size in cm						
Median			P = 0.16	4	4.5	P =0.34

Sig, significant; #, Fischer’s exact test; Bolded vales, Signiﬁcant. *, Mann-Whitney

**Table 3:** Kaplan Miere Analysis of Clinicopathologic Features and CD10 Protein Expression for OS, RFI, and MFS

Parameters	OS		RFS		MFS	
	χ2	P	χ2	P	χ2	P
Sex	0.71	0.39	0.67	0.41	0.69	0.4
Male						
Female						
Grade	6.2	**0.013**	9.5	0.002	2.34	0.13
Low						
High						
Stage	18.97	**0.002**	22.5	**<0.001**	5.7	0.33
pTa						
pT1						
pT2a						
pT2b						
pT3						
pT4						
Bilharziasis	0.025	0.875	1.62	0.2	0.32	0.57
Present						
Absent						
Squamous differentiation	3.39	0.65	4.23	**0.04**	1.29	0.26
Present						
Absent						
Lymphovascular invasion (LVI)	14.7	**<0.001**	24.36	**<0.001**	5.6	**0.018**
Present						
Absent						
Lymph node metastasis (LNM)	12.59	**< 0.001**	6.39	**0.011**	4.7	**0.03**
Present						
Absent						
Perineural invasion (PNI)	11.05	**0.001**	11.8	**0.001**	1.7	0.189
Present						
Absent						
Necrosis	0.78	0.38	4.15	**0.042**	0.58	0.44
Present						
Absent						
CD10 expression in tumor	4.24	**0.04**	7.05	**0.008**	0.25	0.62
Positive						
Negative						
CD10 expression in stromal fibroblast	8.099	**0.004**	9.79	**0.002**	3.23	0.72
Positive						
Negative						

Sig, significant; Bolded vales, Signiﬁcant.

**Table 4:** Correlation between Clinicopathologic Features and CD10 (MME) mRNA in Tumor Using qRT-PCR

Parameters	Median fold change	p
Age		
< 50 Years	7.494	P=0.35
≥ 50 Years	6.185	
Sex		
Male	6.265	P=0.761
Female	6.967	
Grade		
Low	1.874	**P<0.001**
High	7.857	
Stage		
pTa	1.133	P<0.001*
pT1	3.185	
pT2a	4.442	
pT2b	6.795	
pT3	8.894	
pT4	10.04	
Papillary architecture		
Present	3.776	**P<0.001**
Absent	8.681	
Bilharziasis		
Present	7.246	P=0.975
Absent	6.185	
Squamous differentiation		
Present	6.876	P=0.548
Absent	6.356	
LVI		
Present	8.456	P <0.001
Absent	2.591	
LNM		
Present	7.857	P=0.214
Absent	6.265	
PNI		
Present	8.338	P <0.001
Absent	2.939	
Necrosis		
Present	7.494	P=0.051
Absent	3.926	
Recurrence		
Present	8.907	**P=0.01**
Absent	5.113	
Metastasis		
Present	10.996	P=0.283
Absent	6.265	
Living Status		
Alive	6.184	P=0.12
Dead	9.172	

Data was analyzed statistically using MannWhitney U test and Kruskal-Wallis Test; P value was considered significant if ≤ 0.05

**Table 5.** Relationship between CD10 IHC Expression in Both Tumor and Stromal Fibroblasts and CD10 (MME) mRNA in Tumor Using qRT-PCR

Parameters	Median fold change	p
CD10 Expression in tumor cells		
Negative < 5%	1.959	P <0.001
Positive ≥ 5%	7.452	
CD10 Expression in stromal fibroblasts		
Negative < 5%	2.249	P=0.002
Positive ≥ 5%	7.452	
CD10 group expression		
Positive tumor positive stroma	7.495	P=0.007*
Positive tumor Negative stroma	3.926	
Negative tumor Positive stroma	3.773	
Negative tumor Negative stroma	1.325	

Data was analyzed statistically using MannWhitney U test and *Kruskal-Wallis Test; P value was considered significant if ≤ 0.05

**Table 6:** Correlation between CD10 Marker in both IHC and PCR Techniques among Cases Using Spearman’s rho:

	CD10mRNA	CD10 IHC expression in Tumor cells	CD10 IHC expression Stromal cells
CD10mRNA			
correlation coefficient	1	0.487	0.44
sig. (2-tailed)		**<0.001**	**0.002**
CD10 IHC expression in Tumor cells			
correlation coefficient	0.487	1	0.678
sig. (2-tailed)	<0.001		**<0.001**
CD10 IHC expression Stromal cells			
correlation coefficient	0.44	0.678	1
sig. (2-tailed)	**0.002**	**<0.001**	

## Discussion

Despite advances in diagnostic and therapeutic techniques, the outcomes for bladder cancer patients have obviously remained unchanged. The management of bladder cancer still depends on pathologic staging, which not always reflects the actual risk for each patient. Therefore, the trend now for assessing molecular alterations in individual tumors, thus offered detection of various cellular pathways that are deregulated in bladder tumorigenesis and progression (Zlatev et al., 2015) .

With regard to the stage of the tumor, pT3 (44.3%) was the most prevalent stage in our study which was correlated with finding found in an Egyptian study done by Abdou ( 2007). However, this finding was in contrast with studies done by Bahadir et al., (2009), Mohammed (2013), and Atique et al., (2014), revealing that the commonest stages were pTa (75.2%), pT1 (32%), and pT2 (44%), respectively.

The current study revealed that high grade tumors (84.8%) were more frequent than low grade ones. This finding was in agreement with those reported in studies done by Abdou (2007) and Atique et al., (2014), which were 56% and 46%, respectively. In other study done by Bahadir et al., (2009), low grade UBC (59.8%) was more frequent than high grade one. The difference between Egyptian, Eastern, and Western data could be attributed to the strict early screening programs in Western countries.

In our study, we noticed that normal urothelium was negative for CD10 by IHC staining , while tumor cells stained positive for CD10 . This finding was in keeping with studies done by Abdou (2007), Chu and Arber (2000), and Bircan et al., (2006). Although Koiso et al., (1994) found that CD10 was expressed in normal urothelium, its expression was focal and weak compared to its expression in neoplastic lesions. These findings may suggest that CD10 is expressed in the neoplastic rather than the normal epithelium during tumorigenesis, and it may be involved in neoplastic transformation in the bladder urothelium (Bircan et al. 2006). Murali et al., (2005) demonstrated that diffuse and strong CD10 staining was found in invasive UCB, high grade papillary UCB, and CIS cases, while focal and weak staining was detected in urothelial dysplasia, low grade UCB, and PUNLMP.

*CD10* is widely expressed in the stroma of various tumor types and correlates with poor prognosis (Zhu et al. 2016). In the current investigation, we studied the expression of CD10 protein in both tumor and investing reactive stromal fibroblasts. We found a positive significant correlation between *CD10* expression in either tumor cells or stromal fibroblasts and tumor grade. This was in line with a studies done by Bahadir et al., (2009), Murali et al., (2005), and Omran (2012) . However, Abdou (2007) and Bircan et al., (2006) revealed no significant correlation between *CD10* expression either in tumor or stromal cells and tumor grade.

Our study showed significant association between *CD10* expression in either tumor or stromal cells and tumor stage. This was in agreement with studies done by Abdou (2007) and Omran (2012). However, Bahadir et al., (2009) found an inverse correlation between* CD10 *expression and tumor stage, reporting that higher level of CD10 expression was found in non-invasive carcinomas (Bahadir et al., 2009). 

Our study revealed a significant association between *CD10* expression in either tumor cells or stromal fibroblasts and other clinicopathologic features related to aggressiveness such as LVI and PNI. This finding was incompatible with a study done by Abdou (2007) who found no significant association between *CD10* expression in either tumor or stromal cells and LVI.

We demonstrated no association between *CD10 *expression in tumor cells and squamous differentiation, LNM, and tumor necrosis. This finding matched with that yielded by Abdou (2007), while it was in contrast with findings of a study done by Omran (2012), reporting significant association between CD10 expression in tumor cells and LNM.

Unlike* CD10* expression in tumor cells, *CD10* expression in stromal fibroblasts was significantly associated with squamous differentiation. A previous study revealed that uroyhelial carcinoma with quamous differentiation was associated with worse outcome and was resistant to urothelial carcinoma treatment (Adam and DeGraff, 2015) .

There was also significant association between CD10 expression in stromal fibroblasts and lymph node metastasis as well as tumor necrosis. These findings were in agreement with those reported in the study done by Omran (2012). Zhang et al., (2015) concluded that tumor necrosis was an independent factor of adverse clinical outcomes in node negative.

In this work, no significance correlation was detected between *CD10* expression in either tumor cells or stromal fibroblasts and patient’s age, sex, tumor size, or bilharzial infestation. Similarly, Abdou (2007) found no significant association between *CD10* expression in either tumors or stromal cells and different clinicopathologic data except for tumor size. Omran (2012) showed that the frequency of stromal expression of *CD10* was significantly higher in bilharzial-associated bladder carcinomas than in nonbilharzial, which was in contrast with our findings.

With regard to survival analysis, we observed that high tumor grade, advanced stage, LVI, LNM, PNI, and positive *CD10* expression in both tumor and stromal cells were associated with shorter OS. Few parameters in agreement with series done by Abdou (2007), who concluded that tumor grade, CD10 tumor cells expression, and stromal *CD10* expression were associated with significant poor survival. This discrepancy could be attributed to the number of cases involved in our study which was larger (106 cases) compared to 49 cases in his study. urinary tract urothelial carcinoma patients. 

Regarding RFS, we found that high tumor grade, advanced stage, LVI, LNM, PNI, tumor necrosis, squamous differentiation, and positive CD10 staining in both tumor cells and stromal fibroblasts were associated with shorter RFS. In addtion, we noticed that LVI and LNM were associated with shorter MFS. Muppa et al., (2017) emphasized that lymphovascular tumor emboli should be considered carefully as it contributes to early metastasis and worse outcome.

Global *mRNA* expression analysis is accurate for phenotypic profiling of tumors, and also has been used to define molecular subtypes for different tumor types (Marisa et al., 2013, Sjodahl et al., 2017). A key limitation is that most tumors are composed of both tumor and non-tumor cells. This problem is particularly relevant for analysis of advanced invasive tumors, which are known to induce major changes and responses in both tumor and its surrounding tissue (Sjodahl et al., 2017). To minimize this limitation, in this study, we examined the expression of CD10 protein in both tumor cells and investing reactive fibroblasts by using IHC protocol, and then compared the expression of CD10 mRNA in tumor specimen by using RT-PCR.

*CD10 mRNA *expression was examined in different tumor types in the literature. One of them studied CD10 and other ectopeptidases upregulation in gastric cancer (Carl-McGrath et al., 2004). Moreover, *CD10 mRNA *expression in hepatocellular carcinomas was studied by using RT-PCR and it was revealed that expression of CD10 in non-neoplastic and neoplastic hepatocytes correlated inversely with their state of proliferation or differentiation (Rocken et al., 2004). Quantification of CD10 mRNA in colorectal cancer and the relationship between mRNA expression and liver metastasis was analyzed in one study. In aforementioned study, It was noticed that CD10 mRNA was significantly higher expressed in tumor tissues than in matched normal tissues, and CD10 mRNA was associated with invasion depth, lymph node status, and TNM stage (Fujitaet al., 2007).

The results on CD10 IHC staining in our study was consistent with those in most of previous studies investigated *CD10 IHC* expression in urothelial carcinoma (Abdou, 2007; Bahadir et al., 2009; Atique et al., 2014), thus prompting us to examine the association between *CD10 mRNA* expression level in urothelial carcinoma and patients’ clinicopathologic parameters and its impact on prognosis using RT-PCR. To our knowledge, this study was the first one in literature quantifying CD10 mRNA in urothelial carcinoma of bladder using qRT-PCR. In this study, we also examined the relationship between CD10 mRNA and different clinicopathologic data. 

The current study demonstrated that the mean of CD10 mRNA in tumor (mean ∆CT=11.82) was higher than that in matched normal tissues (mean ∆CT=8.88), which was in line with findings reported by Fujitaet al., (2007) on colon cancer. They yielded that CD10 mRNA in tumor tissues was higher than that in matched normal tissues. 

*CD10 mRNA* expression in tumor was significantly associated with tumor grade and stage. These outcomes coincided with Fujita et al’s (2007) findings indicating that the level of CD10 mRNA was higher in advanced tumor stage and high tumor grade. These findings suggested that CD10 mRNA level was associated with tumor progression.

Many studies suggested the role of CD10 in tumor progression (Bilalovic et al., 2004; Iwase et al., 2005; Abdou, 2007; Lee et al., 2015) and metastasis (Huang et al., 2005; Abdou, 2007; Ohji et al., 2007). In this study, there was a significant correlation between *CD10* tumor expression and LVI, PNI, and tumor recurrence. Despite that *CD10 mRNA *expression was higher in metastatic group than in non-metastatic one, there was no significant association between *CD10 mRNA *expression and distant metastasis. These results may be due to small sample size comparing to larger one in IHC study .

Given that CD10 mRNA was extracted from both tumor and stroma in the present study, it was quantified in both tissues as a whole and the relationship between CD10 mRNA of tumor specimen and CD10 IHC of both tumor cells and stromal fibroblast were tested. The highest fold change was present in cases with coincide expression of *CD10 IHC* in both tumor and stromal cells.

Correlation between CD10 mRNA of tumor and CD10 IHC of both tumor cells and stromal fibroblast was examined by Spearman’s rho. A strong positive correlation was found between *CD10 mRNA* and *CD10 IHC* expression in tumor cells and stromal fibroblasts.

In conclusion, increased expression of *CD10* in the tumor and stromal cells was strongly correlated with tumor progression, invasion, metastasis, shorter OS, and RFS in urothelial carcinoma patients.

CD10 mRNA showed significantly higher expression in tumor tissue than in matched normal tissue. High *CD10 mRNA* expression was associated with depth of invasion, advanced stage, high tumor grade, vascular tumor invasion, and recurrence. Therefore, it seems that CD10 mRNA extracted from tumor tissues might be useful as a predictor of tumor recurrence and as a prognostic marker. *CD10 mRNA* expression in urothelial carcinoma was neither associated with distant metastasis nor with lymph node metastasis, thus it cannot be predictor of either nodal or distant metastasis.

The results of IHC and RT-PCR were almost matched. Therefore, we can depend on IHC technique for investigating* CD10* expression, as it is easier and cheaper compared to RT-PCR technique. RT-PCR technique can be done on stored formalin fixed paraffin embedded blocks with accurate results, allowing us to use it for advanced molecular study and correlate with archived data to achieve appropriate therapy .

## Conflict of interest

None.
